# Differentiation of Phenolic Composition Among Tunisian *Thymus algeriensis* Boiss. et Reut. (*Lamiaceae*) Populations: Correlation to Bioactive Activities

**DOI:** 10.3390/antiox8110515

**Published:** 2019-10-28

**Authors:** Rym Jaouadi, Artur M. S. Silva, Mohamed Boussaid, Imen B. H. Yahia, Susana M. Cardoso, Yosr Zaouali

**Affiliations:** 1 Laboratory of Plant Biotechnology, Department of Biology, National Institute of Applied Science and Technology, B.P. 676, Tunis CEDEX 1080, Tunisia; jaouadi.rima@gmail.com (R.J.); mohamed.boussaid@insat.rnu.tn (M.B.); imenbenhajyahia@gmail.com (I.B.H.Y.); 2 QOPNA & LAQV-REQUIMTE, Department of Chemistry, University of Aveiro, 3810-193 Aveiro, Portugal; artur.silva@ua.pt

**Keywords:** thyme, UHPLC-DAD-ESI/MS^n^, phenolic compounds, antioxidant activity, anti-acetylcholinesterase activity, antibacterial activity

## Abstract

Twelve Tunisian *Thymus algeriensis* populations growing wild in different bioclimatic zones, extending from the subhumid to the upper-arid bioclimates, were compared regarding their phenolic composition and their ability to serve as antioxidant, anti-acetylcholinesterase, and antibacterial agents. A significant variation of phenol profile was observed between the analyzed populations, as assessed by ultra-high-performance liquid chromatography coupled with a diode array detector and an electrospray mass spectrometer (UHPLC-DAD-ESI/MS^n^) technique. Rosmarinic acid was the main phenolic compound in most populations (383.8–1157.8 µg/mL extract), but still, those from the upper-arid bioclimatic zone were distinguished by the presence of carvacrol (1374.7 and 2221.6 µg/mL extract), which was absent in the remaining ones. *T. algeriensis* methanolic extracts were found to possess a substantial antioxidant and anti-acetylcholinesterase activities, with significant variation observed between populations, which were correlated to their phenolic contents. The antibacterial activity of the extracts tested against seven bacteria was revealed only by populations collected from upper-arid bioclimate and mainly associated with the presence of carvacrol. Extracts revealed a bacteriostatic effect against all bacteria (MIC = 1.4 mg/mL). Yet, the bactericidal activity (MBC = 1.4mg/mL) was restricted to the gram-negative bacteria *Escherchia coli.*

## 1. Introduction

Nowadays, there is great interest in medicinal plants and their bioactive compounds, namely antioxidant compounds, to be used as health-promoting agents in distinct industrial fields. *Thymus* species are aromatic plants known for their richness in bioactive phytochemicals [1], including phenolic acids and flavonoids [2].

In most parts of the world, thyme is considered one of the most valuable spices/food preservatives in the food industry [3]. In addition, it is commonly used in folk medicine because of its medicinal properties, including antispasmodic, antioxidant [4,5,6], antifungal [7,8], antibacterial [9,10,11], antitumor [12] and anti-aflatoxigenic [13] properties.

In Tunisia, the genus *Thymus L*. includes *Thymus capitatus* (L.) Hoffm. et Link., (= *Thymbra capitata* (L.) Cav = *Coridothymus capitatus* (L.) Rchb. F. = *Satureja capitata* L. = *Thymus cephalotus* L), *Thymus numidicus* Poir., *Thymus algeriensis* Boiss. and Reut., (= *Thymus hirtus* subsp. algeriensis (Boiss. et Reut.)), and *Thymus vulgaris* L. [14]. *Thymus algeriensis* is an endemic species of Morocco, Algeria, Tunisia and Libya [14,15] and described to be a diploid (2*n* = 2*x* = 30) and gynodioecious species [16]. In Tunisia, this species has a wide geographical distribution and grows spontaneously in diverse bioclimatic zones, extending from the subhumid to the lower arid bioclimates. It is largely used, fresh or dried, in food, as a culinary herb and in folk medicine, mainly due to its protection against abortion and respiratory and digestive tube disorders [12,17].

The chemical composition and biological properties of essential oils of *T. algeriensis* from different geographical areas have been reported [18,19,20,21]. In turn, to the best of our knowledge, there are only a few studies focusing on the phenolic composition of this species, although previous bibliography highlighted its richness in flavonoids [22,23] and phenolic acids [24], thus suggesting its suitability to serve as a source of bioactive compounds. This work is complementary to that performed by members of our group [16,25] that focused on the genetic variability of *T. algeriensis* with regard to its essential oils. In the present study, variability of phenolic compounds is assessed in twelve Tunisian populations, collected from four different bioclimatic zones, while the potency of their respective methanolic extracts to serve as antioxidant and antibacterial agents, or to inhibit acetylcholinesterase activity, are correlated with their phenolic profile. Please note that the study of the chemical polymorphism in the natural populations of *T. algeriensis* species can provide new insights that may result in the selection of populations with high phenolic/bioactive components to be used by food and pharmaceutical industries [3].

## 2. Materials and Methods

### 2.1. Chemicals

Rosmarinic acid, carvacrol, luteolin-7-*O*-glucoside, kaempferol, apigenin-7-*O*-glucoside, and eriodictyol-7-*O*-glucoside were purchased from Extrasynthese (Genay, France). Methanol, *n*-hexane, and acetonitrile with HPLC purity were obtained from Lab-Scan (Lisbon, Portugal). Water was treated in a Direct-Q^®^ water purification system (Merck Life Science, Darmstadt, Germany). Folin-Ciocalteu reagent was obtained from Panreac (Barcelona, Spain), sodium carbonate (Na_2_CO_3_), aluminum chloride (AlCl_3_), gallic acid, rutin, 2,2-diphenyl-1-picrylhydrazyl (DPPH•), 6-hydroxy-2,5,7,8-tetramethylchromane-2-carboxylic acid (Trolox), butylated hydroxytoluene (BHT), lyophilized acetylcholinesterase (AChE, from electric eel, type VI-S), Iodure acetylthiocholine, DTNB 5,5′-dithio-bis-[2-nitrobenzoic acid] (DTNB), and Donepezil were purchased from Sigma-Aldrich (St. Louis, MO, USA). The 2,4,6-tripyridyls-triazine (TPTZ) and β-carotene were obtained from Fluka (Munich, Germany). Tryptic soy broth was purchased from Scharlau Microbiology (Barcelona, Spain).

### 2.2. Plant Material

Leaves from twelve populations of *T. algeriensis* belonging to different geographic and bioclimatic zones in Tunisia, namely sub humid (Sh), upper semi-arid (Usa), mean semi-arid (Msa), lower semi-arid (Lsa), and upper arid (Ua) (Table 1), were collected during the flowering stage, to assure maximal phenolic amounts and homogeneity among samples (differences between individuals within and between populations are known to be more evident at the vegetative stage). The collected plants were identified by Pr. M. Boussaid from the INSAT (Department of Biology), and voucher specimens were deposited in the Herbarium of the National Institute of Applied Science and Technology of Tunis (T.a. INSAT, 15). From each population, ten individuals at the flowering phase were sampled randomly in an area exceeding 2 ha. Plant materials were dried for 7 days, at room temperature, in the absence of direct sunlight, reaching a final moisture level close to 10%, and then they were powdered by grinding.

### 2.3. Preparation of Extracts

The extracts were prepared following the general procedure previously described [26]. In detail, 1 g of dried leaves of *T. algeriensis* were macerated in 10 mL of methanol for 24 h, at room temperature. Extraction with methanol was reported as the most effective for the recovery of phenolic compounds [27]. The samples were filtered and stored +4 °C for general analysis.

### 2.4. Analysis of Phenolic Compounds

#### 2.4.1. Determination of Total Phenolic and Flavonoid Contents

Total phenolic content was determined using the Folin-Ciocalteu method [28], with some modifications. In more detail, 0.5 mL of diluted sample extract was added to 2 mL of Folin-Ciocalteu reagent, followed by the adding of 2.5 mL of sodium carbonate solution (7.5%) and reading of absorbance at 760 nm, after incubation for 90 min in the dark. Results were expressed as gallic acid equivalents per g of plant dried weight (mg GAE/g leaves DW).

Estimation of total flavonoid content in each extract was performed using the AlCl_3_ method, as reported previously by [26], with minor modifications. One milliliter of each diluted sample was mixed with 1 mL of AlCl_3_ solution (2%), followed by the reading of absorbance at 430 nm after 15 min of incubation. The percentage contents of flavonoids were expressed as mg rutin equivalents per g of plant dried weight (mg RE/g leaves DW).

#### 2.4.2. Identification and Quantification of Phenolic Compounds by UHPLC-DAD-ESI/MS^n^

The individual phenolic compounds of the twelve populations of Tunisian *T. algeriensis* were identified by UHPLC-DAD-ESI/MS^n^ analysis, following the procedure previously described [29], performed on a Ultimate 3000 (Dionex Co., San Jose, CA, USA) apparatus equipped with an ultimate 3000 Diode Array Detector (Dionex Co., San Jose, CA, USA) and coupled with a Thermo LTQ XL (Thermo Scientific, San Jose, CA, USA) ion trap mass spectrometer equipped with an ESI source. The column used was a 100 mm length, 2.1 mm i.d., 1.9 μm particle diameter, end-capped Hypersil Gold C18 column (Thermo Scientific, San Jose, CA, USA), and its temperature was maintained at 30 °C. Quantification of phenolic compounds was performed by peak integration, using the external standard method, with the closest reference compound available [30].

### 2.5. Antioxidant Assays

#### 2.5.1. DPPH• Scavenging Test

The free radical scavenging activity of extracts was measured using the 1,1-diphenyl-2-picryl-hydrazil radical (DPPH•), following the procedure previously described by Zaouali et al. [31]. One milliliter of diluted extract was added to 3 mL of the methanol-DPPH• solution (4 × 10^−5^ M) and stored in the dark. After 30 min, the decrease in absorbance was measured at 517 nm against a blank (methanol solution). Trolox was used as a positive control. Results were expressed as EC_50_ (the efficient concentration to scavenge 50% of DPPH•).

#### 2.5.2. β-Carotene Bleaching Test

The β-carotene method was carried out according to [31]. The absorbance was measured at 470 nm. The same procedure was repeated with the synthetic antioxidant butylated hydroxytoluene (BHT) as a positive control. Results were expressed as EC_50_. An extract concentration providing 50% inhibition (EC_50_) was obtained, plotting inhibition percentage versus extract solution concentrations.

#### 2.5.3. Ferric-Reducing Antioxidant Power (FRAP) Assay

The ferric-reducing ability of extracts was determined as described by Zaouali et al. [31]. The FRAP reagent was freshly prepared by mixing acetate buffer (300 mM, pH 3.6), TPTZ solution (10 mM TPTZ in 40 mM HCl), and FeCl_3_-6H_2_O (20 mM) in a ratio of 10:1:1. To perform the assay, 900 μL of FRAP reagent was mixed with 90 μL distilled water and 30 μL of the diluted samples. The absorbance was measured at 593 nm, using FRAP working solution as a blank. A standard curve was prepared using different concentrations of FeSO_4_.6H_2_O. Results were expressed in mmol Fe^2+^/L of extract.

### 2.6. Acetylcholinesterase Inhibition Assay

The anti-acetylcholinesterase activity was assayed by the spectrophotometric method of Elden et al. [32], with some modifications. Then, 20 μL of methanolic extracts (at different concentrations) was mixed with 25 μL of the enzyme solution (0.28 U/mL). After incubation during 15 min at 37 °C, the reaction was then initiated with the addition of 100 μL of acetylcholine solution (0.15 mM), and 500 μL of 0.3 mM 5,5-dithiobis-2-nitrobenzoic acid was added to 355 μL of Tris-HCl buffer (50 mM, pH 8.0, containing 0.1% bovine serum albumin. Results were expressed as EC_50_ (concentration providing 50% of AChE inhibition). Donepezil was used as a positive control.

### 2.7. Antibacterial Activity

#### 2.7.1. Bacterial Strains

The antibacterial activity of methanolic extracts was evaluated against 7 standards bacteria, namely *Staphylococcus aureus*, *Streptococcus feacalis*, *Bacillus cereus*, *Staphylococcus epidermis* (gram-positive), and *Pseudomonas aeruginosa*, *Escherichia coli*, and *Klebsiella pneumonia* (gram-negative). Bacterial strains were cultured overnight at 37 °C in nutrient broth (Scharlau Microbiology, Barcelona, Spain). 

#### 2.7.2. Well-Diffusion Method

Antibacterial tests were carried out via the well-diffusion method [33], using 100 µL of suspension of the tested bacteria, containing 10^5^ CFU/mL of bacterial strains spread on Tryptic soy agar. Then, 70 µL of methanolic extracts were introduced into the well. The inoculated plates were incubated for 24 h at 37 °C. After incubation, the diameters of inhibition zones were used as a measure of antibacterial activity. Gentamicin (30 µg/disc) and dimethyl sulfoxide (DMSO) were used as a positive and negative control, respectively.

#### 2.7.3. Determination of Minimum Inhibitory (MIC) and Bactericidal (MBC) Concentrations

The MIC was defined as the lowest concentration of the total extracts that induces no visible growth of bacteria [34]. Referring to results of the MIC assay, the minimum bactericidal concentration (MBC) was determined. Then, 50 µL from each methanol extract, showing growth inhibition zone, was added to 5 mL of Triptic soy agar (TSA) broth tubes and incubated for 24 h at 37 °C. From tubes which showed no growth, 0.1 mL of cells was spread on TSA agar plates. MBCs were determined as the highest dilution at which no growth occurred on the plates.

### 2.8. Statistical Analysis

The analysis of variance (ANOVA procedure), followed by Duncan’s multiple range tests (SAS 9.1.3 program, SAS Institute Inc, Cary, NC, USA), was used to assess the variation of phenol contents and biological activities among populations. The relationship between populations and biological activities was investigated by the Principal Component Analysis (PCA), using the MVSP 3.1 program (Kovach Computing Services, Pentraeth, Wales). The classification of populations according to their phenolic compounds was evaluated using cluster analysis (MVSP program). Correlations between phenolic compounds and their biological activities were carried out with PROC CORR procedure using SAS version 9 (SAS Institute Inc, Cary, NC, USA).

## 3. Results and Discussion

### 3.1. Total Phenol and Flavonoid Contents

The contents and composition of total phenols and flavonoids differ according to genotype, geographical, and ecological factors [35]. In our work, the contents of phenolic compounds of *T. algeriensis* populations were variable (Table 2), with maximum levels found in plants grown in the upper arid bioclimatic zone (32–34 mg GAE/g leaves DW), intermediate values in population Ta 10 from the lower semi-arid bioclimatic zone (17.1 mg GAE/g leaves DW), and lower levels, not exceeding 14.8 mg GAE/g leaves DW, being found in the remaining samples. In a similar trend, total flavonoids assumed maximum amounts in upper arid samples (approximately 10–11 mg ER/g leaves DW), while variable amounts ranging from 3 to 9 mg ER/g leaves DW were found in the lower semi-arid, mean semi-arid, upper semi-arid populations, and sub humid. As compared to previous literature data, the total amount of phenolic compounds herein found were in general superior to those revealed for *T. algeriensis* plants grown in Gafsa, Tamerza, and Kairouan in Tunisia (7.08–8.81 mg GAE/g leaves DW) [23], and, in particular, those of upper arid zone also overcome the ones reported for *T. algeriensis* from Algeria (18.7 mg GAE/g leaves DW) [36].

### 3.2. Characterization of Phenolic Compounds in T. algeriensis Populations

**Figure 1:**
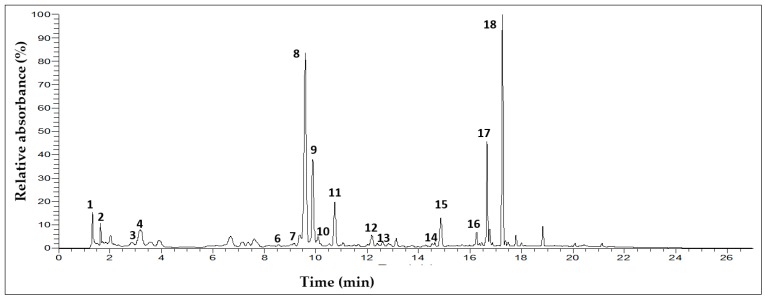
Chromatographic representation of Ta 12 population at 280 nm. Numbers in the figure correspond to the UHPLC-DAD-ESI-MS^n^ peaks described in Table 3.

The individual phenolic components of the distinct *Thymus* populations were elucidated through UHPLC-DAD-ESI-MS^n^ analysis of the respective methanolic extracts. Distinct phenolic compounds were identified among the populations (Table 3, Figure 1). With the exception of Ta 11 and Ta 12 from upper arid rosmarinic acid (peak 8, UV_max_ at 289 sh, and 328 nm, [M − H]^−^ at *m/z* 359) was the main phenolic component identified in the extracts. This is consistent with the general abundance of *Thymus* plants in caffeic acid derivatives, in particular rosmarinic acid [30,37,38,39,40], and also agrees with the recent work of Ziani et al. [24], who showed that, in opposition to aqueous extracts (dominated by flavones), the hydroalcoholic (in that specific case hydroethanolic) extract of a *T. algeriensis* specimen from Algeria was mainly rich in rosmarinic acid. Besides, all the methanolic extracts contained other caffeic derivatives, namely a caffeoyl derivative of rosmarinic acid (eluted in peak 11, [M − H]^−^ at *m/z* 537→493, 359), salvianolic acid K and E ([M − H]^−^ at *m/z* 555 and *m/z* 717 in peaks 10 and 12, respectively), and monomethyl lithospermate (peak 14, [M − H]^−^ at *m/z* 551→519, 359), which are common compounds in *Thymus* plants [2,24,41,42,43,44].

Flavonols were also detected as major phenolic components of *T. algeriensis* methanolic extracts, mostly represented by kaempferol glycosides, particularly kaempferol-*O*-hexoside and kaempferol-*O*-hexuronide (peaks 4 and 6, [M − H]^−^ at *m/z* 447 and *m/z* 461, respectively, and both with UV_max_ at 268 and 339 nm). Interestingly, Ziani et al. [24] also pointed that kaempferol-*O*-hexuronide was a major compound in aqueous or hydroethanolic extracts of Algerian *T. algeriensis*, but, to our knowledge, kaempferol-*O*-hexoside was not previously reported in this species. In regard to the flavone pool, this was represented by luteolin-*O*-hexuronide (([M − H]^−^ at *m/z* 461 and UV_max_ at 255, 266, and 345 nm eluted in peak 5), apigenin-di-*C*-hexoside (peak 2, [M − H]^−^ at *m/z* 593→473, 503, and 575), and apigenin-*O*-hexuronide (peak 6, [M − H]^−^ at *m/z* 445→269), which were all detected before, in the work of Ziani et al. [24]. Moreover, albeit not previously reported, scutellarein (i.e., 4,5,6,7,4’-tetrahydroxyflavone) derivatives were also found (peaks 13, 16, and 17). The first exhibited a pseudo-molecular ion [M − H]^−^ at *m/z* 623 and two fragments at *m/z* 461 and 285, which suggest the loss of a hexose moiety (−162 Da) and the simultaneous loss of hexose and hexuronic units (−162–176 Da), respectively, hence being assigned to a scutellarein-*O*-hexoside-hexuronide. In turn, the compound eluted in peak 16 corresponded to a dimethoxyscutellarein ([M − H]^−^ at *m/z* 313→298), probably cirsimaritin, since this was previously detected in *Thymus* plants [45,46], and the compound eluted in peak 17 ([M − H]^−^ at *m/z* 343→329) was herein tentatively identified as a tetramethoxyscutellarein derivative, based on its MS^2^ fragmentation pattern, which indicated the loss of one to four methyl units (ions at *m/z* 329, 313, 299, and 285).

Like the other described *Thymus* species, the methanolic extracts of *T. algeriensis* also contained flavanones, mainly represented by eriodictyol (peak 7, UV_max_ at 288 and 330sh nm, [M − H]^−^ at *m/z* 287→151, 269) and an *O*-hexoside derivative eluted at RT 2.8 ([M − H]^−^ at *m/z* 449). In addition, samples from upper arid revealed the presence of naringenin (peak 15, UV_max_ at 289, [M − H]^−^ at *m/z* 271→151).

Regardless of the presence of characteristic phenolic compounds from *Thymus* plants in the methanolic extracts of the distinct *T. algerienses* populations (Ta 1–12), specific features were found in some populations. The most striking one was related to the phenolic monoterpene carvacrol, which assumed high levels in samples from the upper arid bioclimatic zone (2222–1375 µg/mL extract for Ta 11 and Ta 12, respectively), standing in contrast to its absence in the extracts from the remaining populations (Table 4). This discrepancy is probably due to environmental and/or genetic factors [47] and also partially justifies the absence of this compound in the work of Ziani et al. [24].

Moreover, high levels of rosmarinic acid were found in population Ta 4 (1157 µg/mL extract) from upper semi-arid bioclimate, followed by populations Ta 10 (1083 µg/mL extract) from lower semi-arid bioclimate and those of the upper arid zones (957 and 807.2 µg/mL extract for Ta 11 and Ta12, respectively), while minimum contents were detected in population Ta 2 (383.8 µg/mL extract) from upper semi-arid bioclimatic zone. As for caffeoyl rosmarinic acid, contents varied between 39.2 µg/mL extract (Ta 1) and 232.2 µg/mL extract (Ta 7), while a moderate average was observed in populations Ta 10 (186.1 µg/mL extract), Ta 11 (206.6 µg/mL extract), and Ta 12 (183 µg/mL extract).

Regarding flavonols, minimum and maximum amounts of the most relevant compound (kaempferol-*O*-hexuronide) were found in populations Ta 3 and Ta 11 (202.9 and 862.80 µg/mL extract, respectively), while kaempferol-*O*-hexoside assumed relevant values in Ta 9 and Ta 5. For the flavone pool, apigenin-*O*-hexuronide was mainly present in population Ta 1 (112.8 µg/mL extract) from the sub humid bioclimate, and it was not detected in populations Ta 11 and Ta 12 from the upper arid zone. Apigenin-*C*-di-hexoside was found in all samples, with values ranging between 10.4 µg/mL extract (Ta 6) and 62.6 µg/mL extract (Ta 9).

**Figure 2:**
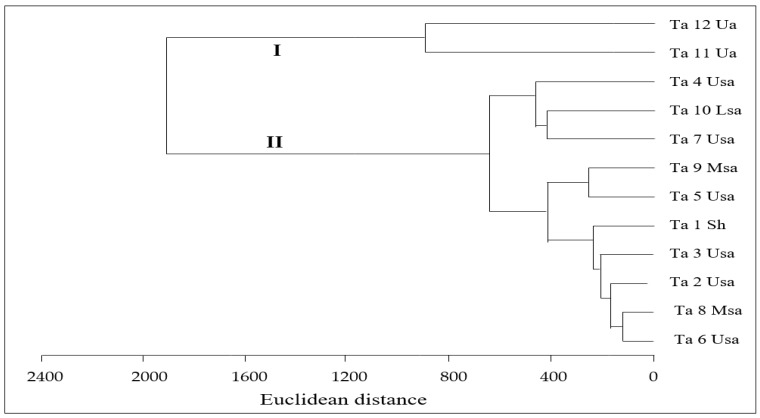
Cluster analysis performed on phenolic compounds of *T. algeriensis* extracts. Ta 1–12 correspond to the code number as detailed in Table 1. Sh, Usa, Msa, Lsa, and Ua correspond to sub humid, upper semi-arid, mean semi-arid, lower semi-arid, and upper arid bioclimatic zones, respectively (See Table 1).

According to their phenolic compounds, the cluster analysis divided the *T. algeriensis* populations in two major groups (Figure 2). The first one (I) was represented by the populations Ta 12 and Ta 11, which were distinguished by their richness in kaempferol-*O*-hexuronide and the presence of carvacrol. The second group (II) was subdivided into two subgroups, one formed by populations Ta 4, Ta 10, and Ta 7, overall characterized by rosmarinic acid abundance, and the other included seven populations (Ta 1, 2, 3, 5, 6, 8, and 9) that were characterized by lower amounts of rosmarinic acid.

### 3.3. Antioxidant Activity

Polyphenols were reported to display several biological effects, including antioxidant activity [30]. The screening of the antioxidant capacity of the methanolic extracts was evaluated by three methods, namely the DPPH• (1,1-diphenyl-2-picryl-hydrazil radical) scavenging assay, β-carotene bleaching test, and the ferric reducing antioxidant power (FRAP).

A significant variation was observed between populations (Table 5), regarding their ability to scavenge DPPH•. In more detail, populations Ta 11 and Ta 12, from the upper arid bioclimatic zones, exhibited the best antiradical capacity (EC_50_ = 8.9 and 10.3 µg/mL, respectively), which was even higher than that of the synthetic compound Trolox.

From the remaining populations, a considerable activity was also observed for populations Ta 10 from lower semi-arid, and Ta 4 and Ta 7 from the upper semi-arid (EC_50_ values of 19.9, 22.7, and 26.6 µg/mL, respectively), while Ta 2 and Ta 3 from the same bioclimatic zone had low scavenging activity (54.5 and 52.3 µg/mL, respectively). Less-promising results were revealed by Ziani et al. [20] for infusion, decoction and hydroethanolic *T. algeriensis* extracts (EC_50_ 48–131 µg/mL), and even reported by Nickavar and Esbati. [48] for other *Thymus* species (31.47–48.68 µg/mL), which exhibited DPPH• EC_50_ values lower than those of the reference commercial compounds.

Notably, *Thymus* populations from the upper arid zone were also the most efficient regarding the potential to protect β-carotene from bleaching. The same trend was found in FRAP assay, with values of 16.7 and 20.6 mmolFe^2+^/L found for populations Ta 11 and Ta12, contrasting with those of the remaining samples (lower than 7 mmolFe^2+^/L).

### 3.4. Anti-Acetylcholinesterase Activity

Phenolic compounds are also claimed to modulate intracellular events involved in distinct neurodegenerative diseases, including the inhibition of AChE, i.e., a central therapeutic target in Alzheimer’s disease [49]. To our knowledge, to the present, only a few studies investigated the ability of *Thymus* extracts in modulating the activity of AChE [50,51,52]. *T. algeriensis* methanolic extracts showed moderate ability to inhibit AChE, with significant variations among populations (Table 5). The weakest activity was observed for the extracts of population Ta 3 (EC_50_ = 3 mg/mL) from upper semi-arid bioclimatic zone. An intermediate activity was revealed for the populations Ta 1, 8, and 10, with EC_50_ values ranging from 1 to 1.2 mg/mL, while populations Ta 11 and Ta 12 from the upper arid zone showed the best activity (EC_50_ of 0.2 and 0.1 mg/mL, respectively). Nevertheless, the inhibitory activity was lower than that of Donepezil (EC_50_ = 18 ± 0.1 μg/mL), a specific inhibitor of acetylcholinesterase, used as a positive control. Curiously, Kindl et al. [52] revealed that ethanolic extracts from *T. longicaulis*, *T. pulegioides*, and *T. vulgaris* exhibited had a lower inhibitory activity against AChE when compared to the reference galantamine (EC_50_ values of 0.66–0.67 mg/mL vs EC_50_ = 0.12 μg/mL).

### 3.5. Antibacterial Activity

The in vitro antibacterial activity of the methanolic extracts estimated by the diameter of inhibition also varied significantly among populations. In fact, only samples collected from upper arid bioclimatic zone, characterized by the presence of carvacrol, showed considerable antibacterial activity (Table 6). The highest activity was observed against *E. coli*, with inhibition zones of 14.5 and 13 mm being recorded for populations Ta 11 and Ta 12, respectively. In turn, inhibition zones for gram-positive strains varied between 10 and 14 mm, with the best activities observed for *S. feacalis*.

The bacteriostatic and bactericidal effectiveness of extracts estimated by MIC and MBC are shown in Table 6. Extracts from upper arid revealed a bacteriostatic effect against all bacteria strains (MIC = 1.4 mg/mL). Although, the bactericidal activity (MBC = 1.4mg/mL) was restricted to the gram-negative bacteria *E. coli*.

### 3.6. Correlations between Bioactivity and Phenolic Components

**Figure 3:**
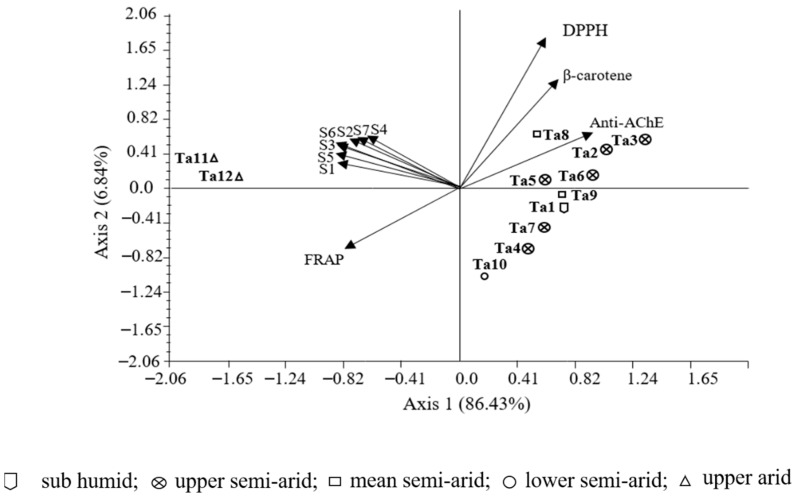
Plot of the principal component analysis performed on values of antioxidant, anti-acetylcholinesterase, and antibacterial activities. Ta 1–Ta 12 correspond to the code number as detailed in Table 1. DPPH•, β-carotene, FRAP: antioxidant assays. S1: *E.coli*, S2: *P. aeruginosa,* S3: *K. pneumoniae*, S4: *S. aureus*, S5: *B. cereus*, S6: *S. epidermis*, S7: *S. feacalis.*

According to axes 1 and 2 (93.27% of the total inertia), the plot of the principal component analysis (PCA) based on the antioxidant, anti-acetylcholinesterase, and antibacterial activities showed two major groups (Figure 3). The first group, at the positive side of axis 1, enclosed ten populations that were less bioactive. The second group, situated at the negative side of axis 1, formed by the two populations Ta 11 and Ta 12 from upper arid bioclimate, characterised by the best antioxidant, anti-acetylcholinesterase, and antibacterial activities.

As expected, significant correlations were observed between total phenol and flavonoid contents and the three antioxidant assays determined by the antiradical activity (*r* = −0.81; *r* = −0.72, *p* < 0.01, respectively), β-carotene bleaching inhibitory activity (*r* = −0.94, *p* < 0.01; *r* = −0.69, *p* < 0.05, respectively), and FRAP assay (*r* = 0.96, *p* < 0.01; *r* = 0.70, *p* < 0.05, respectively) (Table 7). These results are also in line with previous studies that underlined good correlations between polyphenolics and antioxidant activity, due to the effectiveness of these compounds as free-radical scavengers and antioxidants [55].

Regarding specific compositions, DPPH and β-carotene bleaching activities were also correlated to carvacrol (*r* = −0.63; *p* < 0.05, *r* = −0.79; *p* < 0.01), rosmarinic acid (*r* = −0.83, *r* = −0.76; *p* < 0.01), caffeoyl rosmarinic acid (*r* = −0.72, *r* = −0.77; *p* < 0.01, respectively), kaempferol-*O*-hexuronide (*r* = −0.77, *r* = −0.76; *p* < 0.01), and Eriodictyol (*r* = −0.58, *r* = −0.68; *p* < 0.05). Several studies were in accordance with our results revealing free-radical scavenging activity attributed to carvacrol [56,57], rosmarinic acid, and luteolin derivates [38,52,58].

Regarding the anti-acetylcholinesterase activity, a significant correlation was observed between the carvacrol and the EC_50_ values of the enzyme inhibitory (*r* = −0.60, *p* < 0.05) (Table 7). The capacity of the carvacrol as possessing such property was reported by Aazza et al. [59] and Jukic et al. [60]. Besides, our results suggest that other phenolic compounds contribute to the inhibition of the acetylcholinesterase activity. Indeed, a significant correlation was observed between the anti-acetylcholinesterase activity and total phenols (*r* = −0.64, *p* < 0.05) and kaempferol-*O*-hexuronide (*r* = −0.58, *p* < 0.05). 

## 4. Conclusions

This study was carried out in order to describe the phenolic composition of twelve Tunisian *T. algeriensis* populations harvested in different geographical and bioclimatic zones, and to evaluate their biological activities. Rosmarinic acid seems to characterize *T. algeriensis* species, although significant variations in its levels, as well as in other major phenolic compounds, were found. Moreover, populations collected from the most arid zones were characterized by the highest phenolic and flavonoid contents and distinguished by the presence of carvacrol, which was absent in the remaining populations.

All extracts revealed substantial antioxidant activity, as well as anti-acetylcholinesterase and antibacterial activities. The variation of chemical and biological activities among the populations should lead to the selection of plants collected from the most arid zone, with a high potential of antioxidant, anti-acetylcholinesterase, and antibacterial activities, in order to use them in health-care and food industries.

## Figures and Tables

**Table 1:** Main ecological traits of the investigated *T. algeriensis* populations.

Populations	Code	Bioclimatic Zone ^a^	Rainfall (mm/year)	Latitude	Longitude
Korbous	Ta 1	Sub humid (Sh)	550	36°50′	10°35′
Essabahia	Ta 2	upper semi-arid (Usa)	450	36°36′	10°10′
Dj Mansour	Ta 3		450	36°17′	9°36′
Jendouba	Ta 4		660	36°25′	8°44′
Dj chahid	Ta 5		520	36°22′	9°18′
Makther	Ta 6		520	35°51′	9°12′
Kesra	Ta 7		520	35°48′	9°21′
Siliana	Ta 8	mean semi-arid (Msa)	520	35°51′	9°12′
Sers	Ta 9		245	36°6′	9°40′
Sousse	Ta 10	lower semi-arid (Lsa)	167	35°30′	10°50′
Toujene	Ta 11	upper arid (Ua)	100	33°27′	10°08′
Matmata	Ta 12		100	33°32′	9°58′

^a^ Bioclimatic zones were defined according to Emberger’s (1966) pluviothermic coefficient.

**Table 2:** Total phenolic compounds (mg GAE/g leaves DW) and total flavonoid (mg ER/g leaves DW) contents of *Thymus algeriensis*.

	Sh		Usa							Msa			Lsa		Ua	
	Ta 1		Ta 2	Ta 3	Ta 4	Ta 5	Ta 6	Ta 7		Ta 8	Ta 9		Ta 10		Ta 11	Ta 12
TPC	9.1 ^hi^ ± 0.1		9 ^hi^ ± 0.2	8.0 ^i^ ± 0.1	14.8 ^d^ ± 0.8	12.0 ^ef^ ± 0.3	9.8 ^gh^ ± 0.4	12.9 ^e^ ± 0.3		8.2 ^hi^ ± 0.2	11.1 ^gf^ ± 0.2		17.1 ^c^ ± 0.3		34.4 ^a^ ± 0.8	31.6 ^b^ ± 1
TF	2.8 ^h^ ± 0.1		4.8 ^f^ ± 0.2	7.2 ^e^ ± 0.2	9.3 ^b^ ± 0.1	7.5 ^e^ ± 0.1	7.0 ^e^ ± 0.2	4.0 ^g^ ± 0.2		4.0 ^g^ ± 0.1	7.8 ^cd^ ± 0.1		8.2 ^c^ ± 0.2		10.6 ^a^ ± 0.2	10.3 ^a^ ± 0.3

Ta 1–12 correspond to the code number as detailed in Table 1. Sh, Usa, Msa, Lsa, and Ua: sub humid, upper semi-arid, mean semi-arid, lower semi-arid, and upper arid bioclimatic zones, respectively (See Table 1). TPC: total phenolic compounds, TF: total flavonoids. Numbers in lines followed by the same letter are not significant at *p* > 0.05 (Duncan’s multiple range test).

**Table 3:** Phenolic compounds identified by UHPLC-DAD-ESI-MS^n^ in the methanolic extracts of *Thymus algeriensis*.

Peak	t_R (min)_	λmax (nm)	(*m/z*)	MS^2^ions (*m/z*)	Probable Compound
1	1.4	ND	341	179	Caffeoyl hexoside ^b^
2	1.6	271, 330	593	473, 503, 575	Apigenin-di-*C*-hexoside ^b^
3	2.8	284, 325sh	449	259,287	Eriodictyol-*O*-hexoside ^a^
4	3.2	268, 339	447	285	Kaempferol-*O*-hexoside ^b^
5	5.9	255, 266, 345	461	285	Luteolin-*O*-hexuronide ^b^
6	8.8	267, 343	445	269	Apigenin-*O*-hexuronide ^b^
7	9.2	288, 330sh	287	151, 269	Eriodictyol ^a^
8	9.5	287sh, 328	359	161,179, 224	Rosmarinic acid ^a^
9	9.7	268, 339	461	285	Kaempferol-*O*-hexuronide ^b^
10	10.1	288sh, 324	555	493, 357, 393, 313	Salvianolic acid K ^b^
11	10.7	291sh, 324	537	493, 359	Caffeoyl rosmarinic acid ^b^
12	12.2	289sh, 325	717	519	Salvianolic acid E ^b^
13	13.0	273, 330	623	461, 285	Scutellarein-*O*-hexoside-hexuronide ^b^
14	14.5	298sh, 325	551	519, 359	Monomethyl lithospermate ^b^
15	14.9	289	271	151	Naringenin ^a^
16	16.3	277, 333	313	298, 271	Cirsimaritin ^b^
17	16.6	283, 331	343	329, 313, 299 285	Tetramethyl-scutellarein ^b^
18	17.3	276	ND	-	Carvacrol ^a^

ND: not detected. ^a^ Compound identification was based on comparison to standard. ^b^ Compound identification was based on interpretation of UV spectral and MS data, plus comparison to literature.

**Table 4:** Contents (µg/mL extract) of major phenolic compounds in *Thymus algeriensis* for the selected populations.

Compounds		Sh		Usa							Msa			Lsa		Ua	
		Ta 1		Ta 2	Ta 3	Ta 4	Ta 5	Ta 6	Ta 7		Ta 8	Ta 9		Ta 10		Ta 11	Ta 12
Phenolic acids																	
Rosmarinic acid		531.3 ^g^ ± 0.5		383.8 ^j^ ± 0.5	410.4 ^h^ ±0.7	1157.8 ^a^ ±2.7	593.6 ^f^ ±2.1	410.9 ^h^ ±0.7	756.3 ^e^ ± 0.7		391.3 ^i^ ± 0.5	596.4 ^f^ ± 0.3		1083.2 ^b^ ± 3.5		957.0 ^c^ ± 1.0	807.2 ^d^ ± 3.0
Caffeoyl rosmarinic acid		39.2 ^j^ ± 0.1		64.9 ^h^ ± 0.1	78.9 ^f^ ± 0.1	85.5 ^e^ ± 0.1	73.9 ^fg^ ± 0.2	45.8 ^i^ ± 0.0	232.2 ^a^ ± 0.2		74.3 ^g^ ± 0.1	101.6 ^d^ ± 0.1		186.1 ^c^ ± 0.3		206.6 ^b^ ± 1.1	183.0 ^c^ ± 0.5
Flavanones																	
Eriodictyol hexoside		-^h^		6.3 ^f^ ± 0.1	31.9 ^c^ ± 0.1	40.0 ^b^ ± 0.1	39.1 ^b^ ± 0.1	3.5 ^g^ ± 0.2	5.7 ^f^ ± 0.1		28.7 ^d^ ± 0.1	52.8 ^a^ ± 0.1		-^h^		9.1 ^e^ ± 0.2	6.0 ^f^ ± 1.1
Eriodictyol		4.1 ^h^ ± 0.1		16.9 ^b^ ± 0.1	4.4 ^hg^ ± 0.3	12.4 ^d^ ± 0.7	4.4 ^hg^ ± 0.8	8.2 ^f^ ± 0.1	11.4 ^d^ ± 0.1		1.1 ^i^ ± 0.1	5.5 ^g^ ± 0.2		9.9 ^e^ ± 0.4		14.7 ^c^ ± 0.1	42 ^a^ ± 0.1
Flavonols																	
Kaempferol-*O*-hexoside		-^j^		83.9 ^h^ ± 0.3	228.1 ^d^ ± 0.1	326.3 ^c^ ± 0.2	360.4 ^b^ ± 0.5	-^j^	118.4 ^e^ ± 0.2		95.3 ^g^ ± 0.1	439.6 ^a^ ± 0.3		10.0 ^i^ ± 0.2		-^j^	108.4 ± 0.1 ^f^
Kaempferol-*O*-hexuronide		256.3 ^h^ ± 0.3		363.2 ^f^ ± 1.9	202.9 ^j^ ± 1.7	552.0 ^c^ ± 0.6	213.2 ^ij^ ± 12.7	216.5 ^ij^ ± 0.7	526.4 ^d^ ± 0.5		225.2 ^i^ ± 0.4	446.6 ^e^ ± 9.4		297.1 ^g^ ± 0.3		862.8 ^a^ ± 1.2	655.7 ^b^ ± 2.6
Flavones																	
Luteolin-*O*-hexuronide		-^g^		-^g^	-^g^	25.3 ^b^ ± 0.1	20.6 ^c^ ± 0.1	-^g^	12.7 ^d^ ± 0.1		3.0 ^f^ ± 0.1	27.9 ^a^ ± 0.1		5.7 ^e^ ± 0.1		-^g^	-^g^
Apigenin-*C*-di-hexoside		18.4 ^f^ ± 2.7		10.7 ^g^ ± 0.1	21.7 ^e^ ± 0.1	54.1 ^b^ ± 0.1	53.3 ^b^ ± 0.1	10.4 ^g^ ± 0.1	55.3 ^b^ ± 0.3		53.4 ^b^ ± 0.1	62.6 ^a^ ± 0.1		37.7 ^d^ ± 0.2		40.2 ^c^ ± 0.1	54.2 ^b^ ± 0.1
Apigenin-*O*-hexuronide		112.8 ^a^ ± 0.1		6.8 ^c^ ± 0.1	3.8 ^e^ ± 0.1	6.1 ^d^ ± 0.1	3.5 ^ef^ ± 0.1	9.8 ^b^ ± 0.1	3.8 ^e^ ± 0.1		6.1 ^d^ ± 0.1	3.1 ^f^ ± 0.1		1.4 ^g^ ± 0.1		-^h^	-^h^
Phenolic terpene																	
Carvacrol		-^c^		-^c^	-^c^	-^c^	-^c^	-^c^	-^c^		-^c^	-^c^		-^c^		2221.6 ^a^ ± 2.5	1374.7 ^b^ ± 5.0

Ta 1–12 correspond to the code number as detailed in Table 1. Sh, Usa, Msa, Lsa, and Ua: sub humid, upper semi-arid, mean semi-arid, lower semi-arid, and upper arid bioclimatic zones, respectively (See Table 1). Numbers in lines followed by the same letter are not significant at *p* > 0.05 (Duncan’s multiple range test).

**Table 5:** Antioxidant and anti-acetylcholinesterase activities of *T. algeriensis* methanolic extracts.

Bioactivities		Sh		Usa							Msa			Lsa		Ua	
		Ta 1		Ta 2	Ta 3	Ta 4	Ta 5	Ta 6	Ta 7		Ta 8	Ta 9		Ta 10		Ta 11	Ta 12
Antioxidant activity																	
DPPH• (µg/mL)		42.7 ^c^ ± 2.5		54.5 ^b^ ± 2.1	52.3 ^b^ ± 1.4	22.7 ^fg^ ± 0.9	37.8 ^d^ ± 0.6	40.7 ^cd^± 1.0	26.6 ^f^ ± 1.4		68.8 ^a^ ± 1.0	32.4 ^e^ ± 1.0		19.9 ^g^ ± 1.1		8.9 ^h^ ± 0.1	10.3 ^h^ ± 0.4
β-carotene (mg/mL)		1.43 ^e^ ± 0.0		1.50 ^d^ ± 0.1	1.81 ^a^ ± 0.0	1.04 ^h^ ± 0.0	1.35 ^f^ ± 0.3	1.60 ^b^ ± 0.0	1.13 ^g^ ± 0.0		1.60 ^b^ ± 0.0	1.53 ^c^ ± 0.1		0.40 ^i^ ± 0.0		0.03 ^k^ ± 0.0	0.06 ^j^ ± 0.0
FRAP (mmolFe^2+^/L)		2 ^f^ ± 0.0		1.2 ^gh^ ± 0.0	0.3 ^i^ ± 0.01	4.8 ^d^ ± 0.0	6.8 ^c^ ± 0.0	1.8 ^fg^ ± 0.0	5.1 ^d^ ± 0.0		1.0 ^hi^ ± 0.0	4.0 ^e^ ± 0.0		6.5 ^c^ ± 0.05		16.7 ^b^ ± 0.1	20.6 ^a^ ± 0.2
Anti-acetylcholinesterase (mg/mL)		1.0 ^ef^ ± 0.0		1.3 ^e^ ± 0.1	3.0 ^a^ ± 0.1	0.7 ^g^ ± 0.0	2.3 ^b^ ± 0.1	0.9 ^f^ ± 0.1	2.1 ^c^ ± 0.1		1.0 ^ef^ ± 0.0	1.6 ^d^ ± 0.1		1.2 ^e^ ± 0.0		0.2 ^hi^ ± 0.0	0.1 ^i^ ± 0.0

With the exception of FRAP, values are expressed in EC_50_ values, i.e., sample concentration providing 50% of inhibition. DPPH• scavenging activity: EC_50_ values for the positive control Trolox: EC_50_ = 13 µg/mL; β-carotene bleaching inhibition: EC_50_ values for the positive control BHT: EC_50_ = 29.4 ± 0.02 μg/mL; anti-acetylcholinesterase activity: EC_50_ values for the positive control Donepezil: 18 ± 0.1 μg/mL. Ta 1–12 correspond to the code number, as detailed in Table 1. Sh, Usa, Msa, Lsa, and Ua correspond to sub humid, upper semi-arid, mean semi-arid, lower semi-arid, and upper arid bioclimatic zones, respectively (See Table 1). Numbers in the same line followed by the same letter are not significant at *p* > 0.05 (Duncan’s multiple range test).

**Table 6:** Antibacterial activity estimated by diameter of inhibition (mm), minimum inhibitory concentration (MIC), and minimum bactericidal concentration (MBC) (mg/mL) of *T. algeriensis* extracts.

Bacteria		Ta 11					Ta 12		Gentamicin
	ATCC	Inh Zone	MIC	MBC		Inh Zone	MIC	MBC	
Gram-negative									
*E. coli*	10,536	14.5 ^a^ ± 0.0	1.4	1.4		13.0 ^b^ ± 0.0	1.4	-	18.0 ± 0.0
*P. aeruginosa*	9027	10.0 ^a^ ± 0.5	1.4	-		9.0 ^a^ ± 0.0	1.4	-	12.0 ± 0.0
*K. pneumoniae*	10,031	10.5 ^a^ ± 0.5	1.4	-		10.0 ^a^ ± 0.0	1.4	-	17.0 ± 0.0
Gram-positive									
*S. aureus*	6538	11.5 ^a^ ± 0.5	1.4	-		10.0 ^a^ ± 0.0	1.4	-	21.0 ± 0.0
*B. cereus*	11,778	13.5 ^a^ ± 0.5	1.4	-		11.0 ^a^ ± 0.5	1.4	-	17.0 ± 0.0
*S. epidermis*	12,228	12.0 ^a^ ± 0.0	1.4	-		10.5 ^b^ ± 0.0	1.4	-	27.0 ± 0.5
*S. feacalis*	10,541	14.0 ^a^ ± 0.0	1.4	-		13.0 ^a^ ± 0.5	1.4	-	12.0 ± 0.0

Ta 11 and Ta 12 correspond to samples collected from Toujene and Matmata respectively (Table 1). ATCC: American Type Culture Collection; Inh Zone: Inhibition zone expressed in mm. Numbers in the same line followed by the same letter are not significant at *p* > 0.05 (Duncan’s multiple range test).Results demonstrated that only samples containing carvacrol exhibited inhibition zones. This is in accordance with the work of Petrović et al. [53], who suggested that most of the antimicrobial activity of *Thymus* genus appeared to be associated with high amounts of monoterpenic phenols (e.g., carvacrol). In fact, carvacrol is considered to be a biocidal, resulting in bacterial membrane perturbations that lead to leakage of intracellular ATP and potassium ions and ultimately cell death [54].

**Table 7:** Correlations between phenolic content, antioxidant, and anti-acetylcholinesterase activities.

Compounds		Antioxidant Activity				Anti-AChE
		DPPH^•^	β-Carotene	FRAP		Anti-AChE
Total phenols		−0.81 **	−0,94 **	0.96 **		−0,64 *
Flavonoids		−0.72 **	−0.69 *	0.70 *		−0.37 ^ns^
Phenolic acids						
Rosmarinic acid		−0.83 **	−0.76 **	0.54 ^ns^		−0.42 ^ns^
Caffeoyl rosmarinic acid		−0.72 **	−0.77 **	0.67 *		−0.20 ^ns^
Flavanones						
Eriodictyol hexoside		0.20 ^ns^	0.40 ^ns^	−0.22 ^ns^		0.37 ^ns^
Eriodictyol		−0.58 *	−0.68 *	0.78 **		−0.54 ^ns^
Flavones						
Apigenin-*C*-di-hexoside		−0.38 ^ns^	−0.31 ^ns^	0.39 ^ns^		−0.07 ^ns^
Apigenin-*O*-hexuronide		0.19 ^ns^	0.22 ^ns^	−0.26 ^ns^		−0.08 ^ns^
Luteolin-*O*-hexuronide		−0.18 ^ns^	0.15 ^ns^	−0.11 ^ns^		0.23 ^ns^
Kaempferol-*O*-hexuronide		−0.77 **	−0.76 **	0.78 **		−0.58 *
Kaempferol-*O*-hexoside		0.04 ^ns^	0.33 ^ns^	−0.14 ^ns^		0.44 ^ns^
Phenolic terpene						
Carvacrol		−0.63 *	−0.79 **	0.87 **		−0.60 *

*, ** = significant at *p* < 0.05 and *p* < 0.01, respectively; ns = not significant.
